# Posttraumatic stress symptoms in adolescents and young adults with a chronic somatic disease: a mixed-methods study

**DOI:** 10.1186/s13034-023-00630-x

**Published:** 2023-06-26

**Authors:** Frederike Lunkenheimer, Agnes Mutter, Pauline Vogelmann, Harald Baumeister

**Affiliations:** Department of Clinical Psychology and Psychotherapy, Institute of Psychology and Education, Ulm University, Lise-Meitner-Str. 16, 89081 Ulm, Germany

**Keywords:** Posttraumatic stress symptoms, Pediatric Medical traumatic stress, Chronic somatic illness, Comorbidity, Youth, Qualitative and quantitative analysis, Social Burden, Psychological Burden, Physical impairment, Self-management

## Abstract

**Background:**

Adolescents and young adults (AYA) with a chronic somatic disease (CD) have a 3-fold higher risk of post-traumatic stress disorder (PTSD) than healthy controls. In addition, elevated post-traumatic stress symptoms (PTSS) have a negative impact on CD severity, treatment adherence, health problems and functional impairment. However, a more detailed understanding of this comorbidity is lacking.

**Methods:**

AYA with type 1 diabetes mellitus, juvenile idiopathic arthritis or cystic fibrosis (12–21 years of age) and elevated anxiety and/or depression symptoms, as well as their reference persons (≥ 18 years of age), completed online questionnaires in self- or observer report. The most stressful event related to the CD was reported descriptively. Questionnaires were used to assess PTSS, anxious and depressive symptoms, actual overall health, coping, personal growth and social support. Qualitative content analysis, linear regression models and correlations were used for mixed methods analysis.

**Results:**

According to the reports of *n* = 235 AYA (mean age 15.61; 73% girls) and *n* = 70 reference persons, four categories were identified as the most stressful events due to CD: (1) psychological burden (40% of AYA / 50% of reference persons); (2) CD self-management (32% / 43%); (3) social burden (30% / 27%); and (4) physical impairment (23% / 16%). 37% of AYA reported clinically relevant PTSS due to CD. The best predictors of PTSS severity were anxious-depressive symptoms, emotional coping, personal growth and current overall health (*F*(4, 224) = 59.404, *R²* = 0.515, *p* < .001). Of all categories, psychological (*β* = 0.216, *p* = .002) and social burden (*β* = 0.143, *p* = .031) showed significant association with the severity of PTSS (*F*(4, 230) = 4.489, *R²* = 0.072, *p* = .002). The more categories the most stressful event addressed, the higher was the PTSS symptom severity (*r* = .168, *p* = .010).

**Conclusions:**

Many AYA showed clinically relevant PTSS and reported experiencing stressful events in several areas of life through their CD. The association between the stressful event categories and other variables could help identify AYA with CD who need psychological interventions the most.

**Trial registration:**

: German Clinical Trials Register (DRKS): DRKS00016714, registered on 25/03/2019 and DRKS00017161, registered on 17/09/201.

## Background

On average, 40% of the general population has a chronic somatic disease (CD) [1]. In childhood and adolescence 15% of this group suffer from CD, with an increasing trend in number of cases [2, 3]. Common CD include type 1 diabetes mellitus (T1D), with 310/100,000 individuals at the transition to adulthood [4], cystic fibrosis (CF), with 8/100,000 [5], and juvenile idiopathic arthritis (JIA), with 100/100,000 adolescents [6]. As (young) people with CD often experience functional impairments in school, work, leisure and social activities [7] and physical and psychological impairments in their daily lives [8], it is not surprising that more than 40% of people with CD also have a mental disorder [9]. On average, 11.5% of the children and adolescents with CD meet the criteria for post-traumatic stress disorder (PTSD) [10]. Thus the risk of meeting the criteria for PTSD is 2.7-fold higher than their healthy peers [8].

According to the 5th edition of the Diagnostic and Statistical Manual of Mental Disorders (DSM-5; 11), PTSD is a trauma- and stressor-related disorder caused by a traumatic event and is defined by four characteristic symptom domains including intrusion symptoms, avoidance, negative alterations in mood and cognitions, and alterations in arousal and reactivity, causing clinically significant distress and/or functional impairment. Due to changed diagnostic criteria in the recent DSM-5, i.g. the requirements for the fulfilment of the traumatic event were limited, a threat to physical integrity - and thus a CD diagnosis - no longer counts as a criterion for a traumatic event that serves as the basis for a PTSD diagnosis [11, 12]. An exception for this is when CD is associated with increased mortality [11]. A CD which may be experienced as an aversive event given the experience of diagnosis and medical treatment, may be a trigger for elevated post-traumatic stress symptoms (PTSS), but does not necessarily fulfil a diagnosis of PTSD [13]. In addition, children and adolescents with CD report a higher level of PTSS than community norms or healthy control groups (g = 0.50) [10]. Thereby, similar to PTSD, PTSS show a significant negative impact on CD severity, treatment adherence, health problems, and functional impairment [14–17]. This, and the fact that PTSS are more common than PTSD in adolescents and young adults (AYA) with CD [18] and yet PTSS is underestimated [13], show the relevance of PTSS in CD.

The focus of research on AYA with PTSS have often dealt with cancer [19] or chronic pain [20], as well as drastic life events such as heart attack [21], injury [22] or organ transplant [19]. While the perceived threat to life is particularly high for these medical diagnoses and treatment protocols are often very aversive, CD such as T1D, CF and JIA are also associated with prolonged hospital stays, invasive medical interventions and a potential threat to life [23]. Additionally, research often consider PTSS among parents (and especially mothers) of children with CD [24–26] rather than the patients themselves.

An improved and broadened understanding of the comorbidity of CD and PTSS in AYA through the use of mixed methods (combination of qualitative and quantitative analysis) [27, 28], considering self-report and observer-report as well as demographic and psychosocial parameters (e.g. social support, coping, personal growth, depressive and anxious symptoms) promotes early identification of AYA who require psychological support in addition to CD treatment, which in turn may positively impact the course of CD and treatment.

### Aims

The overall purpose of this study was to obtain detailed information about the most stressful events of CD, the severity of PTSS and their association, and other related variables through reports from AYA with T1D, JIA or CF and their reference person. Hence, the following questions were explored in a sample of AYA with T1D, JIA or CF and comorbid elevated anxiety and/or depression symptoms:

1. Which most stressful events related to the CD were reported by AYA with CD (self-report) and their reference person (observer report)?
2. Is there an association between severity of PTSS in AYA with CD and age, gender, type of CD, anxious depressive symptoms, coping, personal growth, current overall health and social support?
3. Is there an association between categories of the most stressful event of CD and age, gender, type of CD and severity of PTSS in AYA with CD?
4. What are the similarities and differences between self- and observer report in relation to the categories of most stressful events due to CD, severity of PTSS, anxious and depressive symptoms and social support in AYA with CD?

## Methods

### Procedure

The study is based on the baseline datasets of a multicenter randomised controlled trial evaluating the (cost-) effectiveness of guided internet- and mobile-based cognitive behavioural intervention for AYA with CD and comorbid depression and anxiety symptoms (*youthCOACHCD*) [29] and the preceding feasibility study [30]. To avoid bias in the data due to treatment effects, the baseline data were analysed cross-sectionally. The studies were conducted within the framework of the *COACH project* (Chronic conditions in adolescents: implementation and evaluation of patient-centered collaborative healthcare). The studies were approved by the ethics committee of Ulm University (Number 292/18) and a-priori registered at the WHO International Clinical Trials Registry Platform via the German Clinical Trials Register (ID: DRKS00016714, 25/03/2019 and DRKS00017161, 17/09/2019). Written informed consent was obtained from all participating AYA; for participants under 16 years of age, written informed consent was also obtained from both legal representatives.

### Participants

AYA with T1D, JIA and CF aged between 12 and 21 years old and elevated anxiety and/or depression symptoms (Generalized Anxiety Disorder Screener, GAD-7 [31] and/or Patient Health Questionnaire, PHQ-9 [32] score ≥ 7) were eligible for participation in case of available internet access, basic knowledge of German language and providing informed consent for participation. AYA with increased risk of suicidality at screening (PHQ-9 Item 9 > 1) were excluded for ethical and safety reasons and referred to ongoing clinical routine [29, 30].

### Recruitment of participants

In the feasibility study, an open recruitment strategy (social media posts in self-help groups for people with CD, flyers in doctors’ offices and clinics or information to email distribution lists of self-help groups) for AYA living in Germany was used between April 2019 and May 2020 [30]. Interested AYA contacted the study team by email [30]. In the (cost-) effectiveness study, recruitment was from October 2019 to June 2022 [29]. Anxious and depressive symptoms were screened as part of routine clinical practice in hospitals, clinics, doctors’ offices and medical centres all over Germany where AYA with T1D, CF or JIA received medical treatment. These clinical sites were all organised in three well-established German patient registries: the National Paediatric Rheumatologic Database (NPRD) [33], the National Diabetes Registry (DPV) [34] and the Cystic Fibrosis (CF) Registry [35]. Screening data were collected in clinical centres and managed within these patient registries. AYA received feedback on their mental well-being from their relevant healthcare provider. If inclusion criteria were met (GAD-7 [31] and/or PHQ-9 [32] score ≥ 7, without PHQ-9 item 9 > 1), AYA were informed by clinic staff and invited to participate in the study, in addition to standard care (treatment as usual). Furthermore, participants could nominate a reference person to provide information about AYA’s health in the observer report. The reference person had to be 18 years of age or older and had to provide written informed consent as well. For each randomised AYA the respective clinical unit received €230 as financial compensation for its recruitment efforts [29].

### Measures

The surveys were conducted on the secure online platform Unipark [36]. Participants and their reference persons were invited by email. If they did not respond to the invitation, they were reminded by email and phone calls. AYA received €10 for each completed survey as compensation. Table 1 gives an overview of all the measurement instruments used. A detailed description of the measurement tools can be found elsewhere [29].

**Table 1:** Measurement instruments

Variables	Measurement
**Self-report, AYA**	
Currently most stressful event with regard to their CD	Brief description, free text
Symptoms of posttraumatic stress disorder	The Child and Adolescent Trauma Screen (CATS) 7–17, Symptom Scale [37]
Depressive and anxiety symptom severity	Patient Health Questionnaire Anxiety and Depression Scale (PHQ-9 and GAD-7, combined PHQ-ADS) [38]
Current overall health	EuroQol visual analogue scale (EQ VAS) from EuroQol Five-Dimensional Questionnaire - Youth (EQ-5D-Y) [39, 40]
Coping	Coping with a Disease (CODI) [41]
Personal growth	Short version of the Stress-Related Growth Scale (SRGS) adapted to AYA with chronic conditions [42, 43]
Social Support	Sub-scale “Actually received support, recipient” of the Berlin Social Support Scale (BSSS) [44]
**Observer report, reference person**	
Currently most stressful event with regard to the AYA´s CD	Brief description, free text
Symptoms of posttraumatic stress disorder	Child and Adolescents Trauma Screen-Caregiver (CATS-C-D) [37]
Symptoms of anxiety	Screen for Child Anxiety Related Emotional Disorders (SCARED) [45]
Symptoms of depression	Mood and Feelings Questionnaire (SMFQ-P) [46]
Social Support	Subscale “Actually received support, provider” of the Berlin Social Support Scale (BSSS) [44]

### Data analysis

#### Qualitative data analysis

The evaluation of the free text description of the most stressful event due to the CD in the self-report and the observer report was systematically analysed with the MAXQDA Software [47] using qualitative content analysis according to Mayring [48]. The categories were assigned deductively by following six main steps: [1] Based on the current literature and relevant findings, a category system with four categories (physical impairments, CD self-management, social and psychological burdens) was defined [8, 49]. [2] After reviewing the data, a coding guideline with definitions, anchor examples and coding rules for each category was defined. [3] After pilot coding of 20% of the data by two independent raters (FL and PV), the category system was revised. [4] First author coded the free text statements using the revised category system. [5] A second independent coder (PV) repeated the coding process. Finally, the coders discussed conflicting decisions in order to reach consensus. In case of disagreement, a third independent rater with experience in qualitative research (AM) would have contributed to the decision-making process. In addition to the description of the content of the categories, the results are also reported quantitatively as frequencies in percentages.

### Quantitative data analysis

The statistical analyses of the descriptive and quantitative data were generated by using SPSS 28.0 [50]. The demographic data were analysed descriptively. The results were presented as a mean with standard deviation or as a proportion. The association between the predictors age, gender, type of CD, current overall health, coping style, level of social support, level of personal growth, depressive and anxiety symptom severity and the severity of PTSS (sum score) were calculated using multiple or stepwise regression. Variables with more than two manifestations (gender and type of CD) were contrast coded and included in the model as categorical predictors together with the continuous variables. The multiple regression analysis was preceded by a correlation analysis to detect suppression effects.

### Mixed methods

The statistical analyses of the mixed methods were generated by using SPSS 28.0 [50]. In accordance with the transfer design (quantification of qualitative data), we implemented the qualitative categories in our regression models. The association between the dummy-coded qualitative categories and the quantitative predictors age, gender and type of CD was calculated using binomial logistic regression. The association between the qualitative categories as dummy-coded predictors and the quantitative variable severity of PTSS (sum score) was calculated using multiple regression. For the comparison of the data in the self-report and the observer report, the variables clinically relevant PTSS (cut-off), anxious symptoms, depressive symptoms, qualitative categories of most stressful events due to CD and social support were analysed using correlations, Chi^2^ tests or t-tests depending on the distribution.

## Results

### Participants

The sample consisted of *n* = 235 AYA (*n* = 15 from the feasibility study, *n* = 220 from the effectiveness study) and *n* = 70 reference persons *(n* = 11 from the feasibility study, *n* = 59 from the effectiveness study). Detailed sample characteristics are displayed in Table 2.

**Table 2:** Sociodemographic characteristics of the sample

Characteristics	AYA (*n* = 235)	RP (*n* = 70)
	*n* (%)	*n* (%)
Gender^a^		
Female	171 (72.8)	65 (92.9)
Male	58 (24.7)	5 (7.1)
Other	3 (1.3)	
Age^b^, *M* (*SD*)	15.61 (2.16)	45.69 (6.58)
Range	12–21	18–58
Relationship of RP to AYA		
Mother	-	63 (90.0)
Father	-	4 (5.7)
Foster mother	-	1 (1.4)
Sister	-	1 (1.4)
Partner	-	1 (1.4)
Chronic condition		
T1D	177 (75.3)	-
CF	14 (6.0)	-
JIA	44 (18.7)	-

*Note*. *N* = 305. AYA = Adolescents and Young Adults; RP = Reference Person; T1D = Diabetes mellitus Type 1; CF = Cystic Fibrosis; JIA = Juvenile Idiopathic Arthritis

^a^ 3 AYA (1.3%) made no indication. ^b^ 12 RP (17.1%) made no indication

37% of AYA reported clinically relevant PTSS (CATS ages 7–17 [37] cut-off ≥ 21) and as many as 82% reported anxious and/or depressive symptoms (GAD-7 [31] and/or PHQ-9 [32] score ≥ 7). 90% of the reference persons were mothers, 6% fathers and 4% siblings, foster mothers or partners of the AYA. The mean age was *M* = 45.69 (*SD* = 6.58). 47% of the reference persons reported clinically relevant PTSS (CATS [37] cut-off ≥ 21) in the AYA. The internal consistency of the questionnaires used in our sample was between McDonald’s Omega (*ω*) = 0.554 and 0.901. Means, standard deviation and McDonald’s Omega are displayed in Table 3.

**Table 3:** Means and standard deviations

Outcome	AYA (*n* = 235)				RP (*n* = 70)		
	*M*	*SD*	*ω*	*M*		*SD*	*ω*
CATS	17.63	9.89	0.873	18.46		9.95	0.901
PHQ-ADS	17.48	8.57	0.885	-		-	-
SCARED	-	-	-	2.00		1.83	0.554
SMFQ-P	-	-	-	8.76		5.48	0.881
SRGS	14.23	7.14	0.898	-		-	-
BSSS	36.13	6.78	0.884	37.07		5.24	0.753
VAS	65.86	20.52	-	-		-	-
CODI							
Avoidance	10.96	3.99	0.752	-		-	-
Acceptance	21.91	4.67	0.814	-		-	-
Cognitive Palliative	10.93	3.64	0.578	-		-	-
Distance	10.26	3.20	0.669	-		-	-
Emotional Reaction	14.06	4.26	0.756	-		-	-
Wishful Thinking	11.14	3.05	0.792	-		-	-
Overall Rating	3.54	0.935	-	-		-	-

*Note*. AYA = Adolescents and Young Adults; RP = Reference Person; CATS = Child and Adolescent Trauma Screen (Range: 0–60); PHQ-ADS = Patient Health Questionnaire Anxiety-Depression Scale (Range: 0–48); SCARED = Screen for Child Anxiety Related Emotional Disorders (Range: 0–10); SMFQ-P = Short Mood and Feelings Questionnaire-Parent (Range 0–26); SRGS = Stress-Related Growth Scale (Range: 0–30); BSSS = Berlin Social Support Scales (Subscale: received/ provided social support, Range: 12–48); VAS = visual analog scale of the EuroQol Five-Dimensional Questionnaire – Youth (Range: 0-100); CODI = Coping with a Disease Questionnaire (Range Overall Rating: 1–5)

### Qualitative data analysis

Intercoder reliability was 62% for the pilot coding. After adjusting the category system and the coding rules for the entire dataset, the intercoder reliability was 76%, with a final consensus of 100%. The four categories were identified as the most stressful events in relation to the CD: *physical impairments, CD self-management, social* and *psychological burdens*. 23% (*n* = 54) of AYA and 16% (*n* = 11) of reference persons reported *physical impairments* such as physical symptoms due to CD, pain in daily life, side effects of medication. 32% (*n* = 74) of the AYA and 43% (*n* = 30) of the reference persons reported burdens from *CD self-management* such as controlling medical parameters in daily life, treatment of CD, medical appointments, taking medication and its organization in daily life. The category of *social burdens* included reports of ignorance or non-acceptance of CD by others, bullying or increased pity and special role due to CD reported by 30% (*n* = 71) of AYA and 27% (*n* = 19) of reference persons. 40% (*n* = 93) of AYA and 50% (*n* = 35) of reference persons reported *psychological burdens* such as lack of acceptance of the CD, fear of the future, fear of dying early, self-doubt, guilt, worry, and loss of light-heartedness. 28.1% (*n* = 66) of AYA and 38.6% of reference persons (*n* = 27) reported more than one category. 8% (*n* = 19) of AYA and 6% (*n* = 4) of the reference persons reported that there were no burdens due to the CD. Example reports on the categories can be found in Table 4.

**Table 4:** Exemplary quotes from AYA and their RP on most stressful events related to the CD

Perceived Challenges	AYA (*n* = 235)		RP (*n* = 70)	
	*n* (%)	Example quotes	*n* (%)	Example quotes
Psychological burdens	93 (39)	“Worries that won’t go away. Fear of not living long. Self-doubt.“ “(…) The look of uncertainty in the future due to the illness, and potential opportunities that will be taken away from me (…)”	35 (50)	“Death. Fear that my child is suffering. Medication administration, by that I mean his quality of life, which he doesn’t have at that moment, but needs to continue living.“ “Daily taking of tablets, with literal disgust for them. Uncertainty of renewed attacks of rheumatism. (…) Sudden panic attacks due to presumably loss of control over the situation.“
Physical impairments	54 (23)	“Fatigue and poor concentration with fluctuating blood glucose levels. Two blood glucose derailments.“ “(…) I can’t dance at parties as long as I would like without having very severe pain in my joints. I have a lot of problems concentrating in school because my pain often makes me lose energy or distracts me. (…)”	11 (16)	“Severe hypos with loss of speech or vision.“ “Glaucoma surgery, visual deterioration, delayed physical development possibly due to medication”
Social burdens	71 (30)	“In terms of my entire diabetes time, what bothered me the most was that a lot of people are uneducated about diabetes and often say stupid things.“ “My father’s little acceptance of having a sick child (…)”	19 (27)	“Handling hyperglycemia and hypoglycemia in society. When she e.g., must eat at school during lessons and thus must constantly discuss with new teachers. Acceptance of her illness by classmates” “(…) feels excluded, in free time and in school”
Self- management	74 (32)	“What stresses me the most is that I always have to have everything with me, that I have to permanently wear an insulin pump and a sensor. Also that I can’t just eat anything, but have to calculate everything. (…)” “Daily time spent not forgetting anything, being constantly reminded not to forget anything (…)”	30 (43)	“That she constantly has to start calculating and counting carbohydrates every time she eats and can never leave the house without her injections (…) and has to control herself all the time” “(…) every 5 days setting the medication injection”

*Note*. AYA = Adolescents and Young Adults, RP = Reference Person. The percentages do not add up to 100% because some AYA and RP have entered multiple categories. *n* = 4 RP (5.7%) and *n =* 19 AYA (8%) reported no burden due to the CD

### Quantitative data analysis

The model for symptom severity of PTSS is statistically significant (*F*(14, 213) = 16.771; *p* < .001), with *R²* = 0.542 (corrected *R²* = 0.509). The predictors PHQ-ADS (*b* = 0.439, *p* < .001), VAS (*b* = − 0.058, *p* = .022), SRGS (*b* = 0.153, *p* = .033), CODI Cognitive-Palliative subscale (*b* = 0.334, *p* = .018) and CODI Emotional Reaction subscale (*b* = 0.807, *p* < .001) are significantly associated with PTSS severity (see Table 5).

**Table 5:** Association between PTSS severity and demographic and psychosocial predictors

Predictors	*b*	*SE*	Β	*t*	*p*	95% CI for *b*	
						*Q1*	*Q3*
Age	− 0.126	0.221	− 0.028	− 0.571	0.569	− 0.562	0.310
Gender							
Male^a^	0.434	1.156	0.019	0.375	0.708	-1.845	2.712
Type of CD							
CF^b^	1.232	1.926	0.030	0.640	0.523	-2.565	5.029
JIA^b^	1.133	1.300	0.045	0.871	0.385	-1.430	3.697
PHQ-ADS	0.439	0.064	0.378	6.863	< 0.001*	0.313	0.565
VAS	− 0.058	0.025	− 0.123	-2.314	0.022*	− 0.107	− 0.009
SRGS	0.153	0.071	0.112	2.150	0.033*	0.013	0.294
BSSS	− 0.076	0.075	− 0.051	-1.013	0.312	− 0.223	0.072
CODI							
Avoidance	0.192	0.141	0.079	1.364	0.174	− 0.086	0.470
Acceptance	− 0.045	0.158	− 0.022	− 0.286	0.776	− 0.357	0.267
Cognitive-Palliative	0.334	0.140	0.125	2.377	0.018*	0.057	0.611
Distance	0.009	0.169	0.003	0.053	0.958	− 0.325	0.343
Emotional Reaction	0.807	0.151	0.352	5.343	< 0.001*	0.510	1.105
Wishful Thinking	− 0.279	0.181	− 0.088	-1.542	0.125	− 0.635	0.078
Overall Coping	− 0.484	0.733	− 0.046	− 0.659	0.510	-1.929	0.962
(Constant)	4.450	6.362		0.699	0.485	-8.091	16.990

*Note*. *N* = 229. Multiple Regression, *R²* = 0.542; corr. *R²* = 0.509; *F*(15, 213) = 16.771; *p* = < 0.001. CI = Confidence interval; CD = Chronic somatic disease; T1D = Diabetes mellitus type 1; CF = Cystic fibrosis; JIA = Juvenile idiopathic arthritis; PHQ-ADS = Patient Health Questionnaire Anxiety-Depression Scale; VAS = visual analogue scale of the EuroQol Five-Dimensional Questionnaire – Youth; SRGS = Stress-Related Growth Scale; BSSS = Berlin Social Support Scales (subscale: social support received); CODI = Coping with a Disease Questionnaire

^a^ Reference category = female. ^b^ Reference category = T1D.

* significant (p < .05)

As a result of stepwise regression (forward selection; see Table 6), the predictors PHQ-ADS (*b* = 0.435, *p* < .001), CODI Emotional Reaction subscale (*b* = 0.894, *p* < .001), SRGS (*b* = 0.214, *p* =. 001) and VAS (*b* = − 0.074, *p* = .002) best predicted PTSS severity, *F*(4, 224) = 59.404, *p* < .001. The model has a high quality of fit with an *R²* = 0.515 (*corrected R²* = 0.506) [51].

**Table 6:** Stepwise regression to analyses the association between severity of PTSS and predictors

Model	*B*	*SE*	Β	*P*	*R²*	Δ*R*	*p*
Model 1					0.326	0.326	< 0.001*
CODI – Emotional Reaction	1.308	0.125	0.571	< 0.001*			
(Constant)	− 0.934	1.845		0.613			
Model 2					0.468	0.142	< 0.001*
CODI – Emotional Reaction	0.977	0.119	0.426	< 0.001*			
PHQ-ADS	0.469	0.060	0.404	< 0.001*			
(Constant)	-4.322	1.699		0.012*			
Model 3					0.493	0.025	0.001*
CODI – Emotional Reaction	0.965	0.117	0.421	< 0.001*			
PHQ-ADS	0.472	0.059	0.407	< 0.001*			
SRGS	0.214	0.065	0.157	0.001*			
(Constant)	-7.282	1.890		< 0.001*			
Model 4					0.515	0.022	0.002*
CODI – Emotional Reaction	0.894	0.116	0.390	< 0.001*			
PHQ-ADS	0.435	0.059	0.375	< 0.001*			
SRGS	0.214	0.063	0.157	0.001*			
VAS	− 0.074	0.023	− 0.157	0.002*			
(Constant)	− 0.760	2.760		0.783			

*Note*. *N* = 229. Stepwise Regression, Model 4: *F*(4, 224) = 59.404, *p* < .001. CODI = Coping with a Disease Questionnaire; PHQ-ADS = Patient Health Questionnaire Anxiety-Depression Scale; SRGS = Stress-Related Growth Scale; VAS = Visual Analogue Scale of the EuroQol Five-Dimensional Questionnaire – Youth

* significant (p < .05)

### Mixed methods

#### Categories and demographic variables

The category physical impairment yielded a statistically significant regression model (*χ²*[4] = 28.24, Nagelkerke *R²* = 0.176, p < .001). The Hosmer-Lemeshow test showed a high quality of fit, χ²[7] = 2.53, *p* = .925. The predictor JIA was significant (95% *CI* [3.166, 15.295], *p* < .001) with an OR = 6.96. The odds of physical impairment were seven times greater for AYA with JIA than for AYA with T1D, holding the other predictors constant. For the CD self-management category, the regression model was statistically significant, *χ²*[4] = 16.85, Nagelkerke *R²* = 0.099, *p* = .002. Quality of fit, tested with the Hosmer-Lemeshow test, was high, *χ²*[8] = 7.56, *p* = .478. Age was a significant predictor in the model (*p* = .041) with an OR = 0.86 (95% *CI* [0.750, 0.994]). Each additional year of life decreases the odds of distress in CD self-management by a factor of one, holding the other predictors constant. JIA was also a significant predictor (*p* = .007), with an OR = 0.25 (95% *CI* [0.091, 0.684]). The odds of burden in CD self-management are 0.25 lower for AYA with JIA than for AYA with T1D. The logistic regression models for the categories psychological burden (*χ²*[4] = 3.91, *p* = .418) and social burden (*χ²*[4] = 6.99, *p* = .136) were not statistically significant. For more information see Table 7.

**Table 7:** Association between the categories of most stressful events due to CD and age, type of CD and gender

	*b*	*SE*	*p*	*OR*	95% CI for *OR*		χ²(4)	*p*	*Nagel-kerke R*²
					*Q1*	*Q3*			
Psychological Burden							3.91	0.418	0.023
Age	0.067	0.065	0.303	1.069	0.941	1.215			
Gender									
Male^a^	− 0.367	0.332	0.268	0.693	0.362	1.327			
Type of CD									
CF^b^	− 0.601	0.619	0.331	0.548	0.163	1.845			
JIA^b^	0.049	0.363	0.893	1.050	0.516	2.137			
(Constant)	-1.331	1.030	0.196	0.264					
Physical Impairment							23.24	< 0.001*	0.176
Age	0.049	0.081	0.544	1.050	0.897	1.230			
Gender									
Male^a^	0.097	0.426	0.820	1.102	0.478	2.541			
Type of CD									
CF^b^	0.832	0.635	0.190	2.298	0.661	7.980			
JIA^b^	1.940	0.402	< 0.001*	6.959	3.166	15.295			
(Constant)	-2.562	1.286	0.046	0.077					
Social Burden							6.99	0.136	0.042
Age	0.058	0.069	0.403	1.060	0.925	1.214			
Gender									
Male^a^	− 0.555	0.373	0.137	0.574	0.276	1.194			
Type of CD									
CF^b^	0.279	0.591	0.638	1.321	0.415	4.210			
JIA^b^	0.475	0.370	0.200	1.608	0.778	3.323			
(Constant)	-1.700	1.103	0.123	0.183					
Self -Management							16.85	0.002*	0.099
Age	− 0.147	0.072	0.041*	0.863	0.750	0.994			
Gender									
Male^a^	− 0.205	0.342	0.548	0.814	0.417	1.591			
Type of CD									
CF^b^	− 0.726	0.680	0.285	0.484	0.128	1.832			
JIA^b^	-1.389	0.515	0.007*	0.249	0.091	0.684			
(Constant)	1.795	1.124	0.110	6.019					

*Note*. *N* = 229. Binomial logistic regressions, CI = Confidence interval; CD = Chronic somatic disease; T1D = Diabetes mellitus type 1; CF = Cystic fibrosis; JIA = Juvenile idiopathic arthritis;

^a^ Reference category = female. ^b^ Reference category = T1D.

* significant (p < .05)

### Categories and severity of PTSS

Multiple regression analysis yielded a significant model for severity of PTSS (*F*(4, 230) = 4.489, *p* = .002) with *R*² = 0.072 (*corrected R*² = 0.056). The predictors psychological (*β* = 0.216, *b* = 4,37, *p* = .002) and social burden (*β* = 0.143, *b* = 3.07, *p* = .031) were significant, physical impairment and CD self-management did not contribute significantly to the explanation of the variance (see Table 8).

**Table 8:** Association between severity of PTSS and categories of most stressful events by CD

Categories	*b*	*SE*	*Β*	*T*	*p*	95% *CI* for *b*	
						*Q1*	*Q3*
Psychological burden	4.365	1.378	0.216	3.168	0.002*	1.650	7.079
Physiological impairment	1.434	1.577	0.061	0.909	0.364	-1.674	4.541
Social burden	3.073	1.420	0.143	2.164	0.031*	0.275	5.871
CD self-management	− 0.833	1.499	− 0.039	− 0.556	0.579	-3.787	2.121
(Constant)	14.907	1.344		11.089	< 0.001*	12.258	17.556

*Note*. Multipe Regression. *R²* = 0.072; corr. *R²* = 0.056; *F*(4, 230) = 4.489; *p* = .002. CI = Confidence interval

* significant (p < .05)

There was a significant correlation between the number of categories reported and the severity of PTSS (r = .168, *p* = .010).

### Similarities and differences between self- and observer report

The categories physical impairment (*χ*²[1] = 5.285, *φ* = 0.275., *p* = .022) and social burden (*χ*²[1] = 4.515, *p* = .034, *φ* = 0.254) as well as elevated depressive symptoms (*χ*²[1] = 4.804, *φ* = 0.262., *p* = .028) were reported significantly more often by AYA than by reference persons. The category CD self-management (*χ*²[1] = 4.667, *φ* = 0.258, *p* = .031) as challenge by CD and elevated anxious symptoms (*χ*²[1] = 13.702, *φ* = 0.442., *p* = .<001) were reported significantly more often by reference persons than AYA. For the category psychological burden and the frequencies of a clinically relevant PTSS, there were no significant differences in self- and observer reporting (see Table 9).

**Table 9:** Frequencies and their comparison for the categories of most stressful events due to CD, PTSS, anxious and depressive symptoms in self-report and observer-report

Categories^a^	AYA (*n* = 70)		RP (*n* = 70)		*P*	χ²(1)	Φ
	*N*	%	*N*	%			
Psychological burden	28	40.0	35	50.0	0.626	0.238	− 0.058
Physiological impairment	14	20.0	11	15.7	0.022*	5.285	0.275
Social burden	20	28.6	19	27.1	0.034*	4.515	0.254
CD self- management	25	35.7	30	42.9	0.031*	4.667	0.258
CATS, Cut-Off ≥ 21	25	35.7	33	47.1	0.108	2.580	0.192
GAD-7, Cut-Off ≥ 7 SCARED, Cut-Off ≥ 3	18	25.7	21	30.0	< 0.001*	13.702	0.442
PHQ-9, Cut-Off ≥ 7 SMFQ-P, Cut-Off ≥ 12	24	34.3	20	28.6	0.028*	4.804	0.262

*Note*. AYA = Adolescents and Young Adults; PTSS = Posttraumatic Stress Symptoms; CD = Chronic Somatic Disease; CATS = Child and Adolescents Trauma Screen, clinically relevant PTSS at a cut-off ≥ 21, self- and observer-report; GAD-7 = Generalized Anxiety Disorder − 7, elevated anxious symptoms at a cut-off ≥ 7, self-report; RP = Reference person; SCARED = Screen for Child Anxiety Related Emotional Disorders, elevated anxious symptoms at a cut-off ≥ 3, observer-report; PHQ-9= Patient Health Questionnaire, elevated depressive symptoms at a cut-off ≥ 7, self-report; SMFQ-P = Mood and Feelings Questionnaire, clinically relevant depressive symptoms at a cut-off ≥ 12, observer-report

^a^*n* = 19 AYA (8,1%) und *n* = 4 RP (5,7%) reported that they were not burdened by the CD.

* significant (p < .05)

There was no statistically significant difference between AYA (*M* = 36.89, *SD* = 6.20) and reference persons’ (*M =* 37.07, *SD =* 5.24*)* reports in terms of AYA social support (BSSS, t(138) = − 0.191, Cohen’s *d* = − 0.032, *p* = .848).

## Discussion

The aim of the study was to gain a deeper insight into the most stressful events due to the CD of AYA with JIA, T1D or CF. Based on the results from both self- and oberserver reports, for all three CD social and psychological burdens, physical impairments and CD self-management challenges were reported as most stressful events due to the CD. The present findings are in accordance with prior evidence derived from studies focusing on CD in AYA [8].The stressful events due to the CD have a relevant impact on mental health, which is demonstrated in high PTSS prevalence of 37% (only related to CD) of AYA with CD in this study.

AYA with CD are faced with additional challenges - besides the usual developmental tasks - such as acceptance of CD, fear of health consequences, dealing with pain, social stigma, management and treatment of CD [7, 8, 49]. These challenges were confirmed in the qualitative analysis of the most stressful events due to CD, reflect the possible suffering and burdens and point to possible triggers for PTSS. Although many young people adapt to the challenge of CD, almost one in nine develops PTSD, three times as much as healthy young people [10].

The quantitative results illustrated the possible impact of PTSS in CD. The higher the severity of PTSS reported by the AYA with CD, the stronger was the personal growth, the higher were comorbid anxious-depressive symptoms, the more frequent was the emotional reaction as a coping strategy in dealing with the CD and the lower was the current overall health. This strong association between PTSS and personal growth, may be due to the fact that maturation through a stressful event can only occur when a stressful event (with or without PTSS as a consequence) has been experienced and overcome [52, 53]. The AYA with increased PTSS have a high mental load in general. This association in this study sample may be due to the inclusion criterion of elevated anxious and/or depressive symptoms. Additionally, the high mental load may be due to common vulnerability factors for trauma and stress-related disorders such as anxiety, depression with PTSD [54]; The result confirms that mental disorders occur more frequently cumulative [55]. Emotionally coping with challenges of the CD is reflected in behaviours such as crying or waking up at night thinking about terrible things [41]. These behaviours are also reflected in symptoms (dream related to the traumatic event, persistent negative emotional state) of PTSS [41], which can be explained by the positive association between PTSS and this coping style. Increased use of emotional coping is associated with decreased emotional and social functioning [56]. Overall, the higher the CD burden is experienced, the worse the current health status is perceived. The known effects of PTSS on health problems and functional impairment [14–17] can also be highlighted by these results and point to the multiple effects that should not be underestimated. AYA with PTSS therefore need increased attention. Early recognition of mental comorbidities, psychosocial support and individual counselling on CD self-management could prevent acute complications and early sequelae [57, 58].

The mixed methods analysis showed that physical impairment was reported seven times more often in AYA with JIA than in AYA with T1D. AYA with T1D were more likely to experience CD self-management as a burden. This is consistent with the typical symptoms of the disease and their treatment according to CD. JIA shows physical impairments through pain and stiffness [59] and T1D is characterised by extensive self-management [57]. The younger AYA with CD seem to be more burdened by the self-management of their CD. Developmentally, younger AYA tend to have more present-oriented thought processes, which can lead to difficulties and overload in anticipatory processes and their organisation, which is, however, fundamental to the self-management treatment of CD [60, 61]. Furthermore, the support and control of parents decreases with the age of the AYA, which could reduce the presence of CD self-management in everyday life and thus the burden [62]. However, the transfer of CD self-management autonomy to the AYA may be reflected in poorer medical parameters of the CD [63]. Thus, there is a dilemma between parental support and the possible increase in AYA burden or handing over responsibility to the AYA and possible resulting deterioration in CD. It would therefore be desirable for AYA to professionally supported in the process of independence in the treatment of CD [64].

Social and psychological burdens were found to be associated with higher PTSS. Thus, the most stressful events due to CD in terms of social environment or psychological condition seem to have a strong influence on the development of PTSS. Social factors appear to strongly influence health in adolescence at the personal, family, community and national levels. Safe and supportive families and schools, as well as positive and supportive peers, are essential protective factors for health development [65]. In the absence of these socially supportive factors, there can be negative effects on mental health, and as this study was able to show, also on PTSS. Other stressful events through CD can also be very burdensome and traumatising, but seem to have a minor impact on the development of PTSS in AYA. Overall, it became clear that the more burdens (the more categories) addressed, regarding the most stressful event due to the CD, the higher was the PTSS severity. These findings are consistent with the knowledge that higher trauma exposure is associated with higher (current and lifetime) prevalence of PTSD and with symptom severity in clear dose-response relationships [66].

AYA reported physical impairments and social burdens due to their CD more often than their reference persons did. However, reference persons were more likely to report burdens from the AYA’s CD self-management than the AYA themselves reported about their CD. An explanation could be that reference persons are more likely to experience the burdens of CD self-management and may themselves be burdened by the support [60, 61]. Caregivers who are psychologically distressed may tend to include their experienced burdens in the observer reports [67]. Thus, the reason for the burden is slightly different depending on the perspective. However, the impact of the most stressful events through the CD on mental health is more than evident through both perspectives. In the self-reports, but also in the observer reports, a clinically relevant PTSS due to the CD was reported in almost every second AYA. Early recognition of mental distress would be feasible through the already existing and regular connection to the health system due to the CD. Raising awareness of this comorbidity would therefore be even more effective in order to be able to provide AYA with psychosocial support in addition to medical support when needed.

### Limitations

In order to obtain specific information about the most stressful events of a CD at AYA, it was essential to ask specifically about this type of index trauma. On the one hand, this is a strength of the study; on the other hand, it limits conclusions about other types of trauma, such as experiences of violence or environmental disasters. Another limitation is that a generalisation of the results from the study to all CD types should only be made with caution due to the sample with the specific CD types T1D, JIA and CF and elevated comorbid anxious and/or depressive symptoms. A further limitation of the study is the lack of assessment of the reference person’s psychological symptoms. The psychological burden of the caregiver might have an influence on the observer reports [67] and would be an interesting area for future research. The study design does not allow conclusions to be drawn about causal relationships, whereby it should be noted that PTSS can only develop under the condition of a stressful situation (due to the CD) [11, 37].

## Conclusions

Many AYA show clinically relevant PTSS and report having experienced stressful events in different life domains through their CD. Information on the most stressful events due to CD could be used in clinical practice to adapt practices for AYA and their reference persons to reduce such distressing experiences. The association between the stressful event categories and demographic and psychosocial variables could help identify AYA with CD who are most in need of psychological interventions. These study findings might be used to raise awareness of the comorbidity of CD and PTSS in clinical practice, to identify psychosocial needs in addition to psychological initial screening and medical treatment, and to offer support at an early stage.

## Data Availability

The datasets analysed during the current study are not publicly available to ensure patient protection, but are available from the author Prof. Dr. Harald Baumeister (harald.baumeister(at)uni-ulm.de) on reasonable request.

## Abbreviations

AYA: Adolescents and Young Adults
CD: Chronic Somatic Disease
CF: Cystic Fibrosis
DSM: Diagnostic and Statistical Manual of Mental Disorders
DPV: National Diabetes Registry
JIA: Juvenile Idiopathic Arthritis
NPRD: National Paediatric Rheumatologic Database
PTSD: Post-traumatic Stress Disorder
PTSS: Post-traumatic Stress Symptoms
T1D: Type 1 Diabetes Mellitus
